# Analysis of the Phenotypes in the Rett Networked Database

**DOI:** 10.1155/2019/6956934

**Published:** 2019-03-27

**Authors:** Elisa Frullanti, Filomena T. Papa, Elisa Grillo, Angus Clarke, Bruria Ben-Zeev, Mercedes Pineda, Nadia Bahi-Buisson, Thierry Bienvenu, Judith Armstrong, Ana Roche Martinez, Francesca Mari, Andreea Nissenkorn, Caterina Lo Rizzo, Edvige Veneselli, Silvia Russo, Aglaia Vignoli, Giorgio Pini, Milena Djuric, Anne-Marie Bisgaard, Kirstine Ravn, Vlatka Mejaski Bosnjak, Joussef Hayek, Rajni Khajuria, Barbara Montomoli, Francesca Cogliati, Maria Pintaudi, Kinga Hadzsiev, Dana Craiu, Victoria Voinova, Aleksandra Djukic, Laurent Villard, Alessandra Renieri

**Affiliations:** ^1^Medical Genetics, University of Siena, Italy; ^2^Institute of Medical Genetics, School of Medicine, Cardiff University, Cardiff, Wales, UK; ^3^Pediatric Neurology Unit and Israeli Rett Clinic, Safra Children Hospital, Chaim Sheba Medical Center, Tel HaShomer, Israel; ^4^Sackler School of Medicine, Tel Aviv University, Tel Aviv, Israel; ^5^Neurologia Fundació Sant Joan de Deu, Barcelona, Spain; ^6^Pediatric Neurology, Necker-Enfants Malades Hospital, University Paris Descartes, AP-HP, Paris, France; ^7^Imagine Institute, Inserm U1163, Team Genetics and Pathophysiology of the Development of Cerebral Cortex, Paris Descartes University, Paris, France; ^8^INSERM, U1016, Paris, France; ^9^Institute Cochin, Université Paris Descartes, CNRS, UMR8104, Paris, France; ^10^Laboratoire de Biochimie et Génétique Moléculaire, Hôpital Cochin, Assistance Publique, Hôpitaux de Paris, Paris, France; ^11^Molecular and Genetics Medicine Section, Hospital Sant Joan de Déu, Barcelona, Spain; ^12^Institut de Recerca Pediàtrica Hospital Sant Joan de Déu, Barcelona, Spain; ^13^CIBER-ER (Biomedical Network Research Center for Rare Diseases), Instituto de Salud Carlos III, Madrid, Spain; ^14^Genetica Medica, Azienda Ospedaliera Universitaria Senese, Siena, Italy; ^15^Child Neuropsychiatry, DINOGMI, University of Genova, Genova, Italy; ^16^Istituto Auxologico Italiano, IRCCS, Laboratorio di Citogenetica e Genetica Molecolare, Cusano Milanino, Milan, Italy; ^17^Epilepsy Center, Childhood and Adolescence Neurology and Psychiatry, ASST Santi Paolo Carlo, Department of Health Sciences, University of Milan, Milan, Italy; ^18^Tuscany Rett Center, Ospedale Versilia, 55043 Lido di Camaiore, Italy; ^19^Neurologic Department, Mother and Child Health Care Institute of Serbia “Dr Vukan Cupic”, University of Belgrade, Belgrade, Serbia; ^20^Centre for Rett Syndrome, Department of Paediatrics and Adolescent Medicine, The Kennedy Center, Copenhagen University Hospital Rigshospitalet, Copenhagen, Denmark; ^21^Department of Neuropediatrics, Zagreb Children's Hospital, University of Zagreb, Zagreb, Croatia; ^22^Department of Molecular and Developmental Medicine, University of Siena, Siena, Italy; ^23^All India Institute of Medical Sciences, Genetics Unit, Department of Pediatrics, New Delhi, India; ^24^Child Neuropsychiatry Unit, Azienda Ospedaliera Universitaria Senese, Siena, Italy; ^25^DINOGMI, University of Genova, Genova, Italy; ^26^Department of Medical Genetics, and Szentagothai Research Center, University of Pécs, Medical School, Pécs, Hungary; ^27^Carol Davila University of Medicine, Pediatric Neurology Clinic, Al Obregia Hospital, Bucharest, Romania; ^28^Ministry of Health, Department of Clinical Genetics, Institute of Pediatrics and Pediatric Surgery, Moscow, Russia; ^29^Tri-State Rett Syndrome Center, Montefiore Medical Center, Albert Einstein College of Medicine, New York City, NY, USA; ^30^Aix Marseille University, Inserm, MMG, U1251 Marseille, France; ^31^Department of Medical Genetics, La Timone Children's Hospital, AP-HM, Marseille, France

## Abstract

Rett spectrum disorder is a progressive neurological disease and the most common genetic cause of intellectual disability in females. *MECP2* is the major causative gene. In addition, *CDKL5* and *FOXG1* mutations have been reported in Rett patients, especially with the atypical presentation. Each gene and different mutations within each gene contribute to variability in clinical presentation, and several groups worldwide performed genotype-phenotype correlation studies using cohorts of patients with classic and atypical forms of Rett spectrum disorder. The Rett Networked Database is a unified registry of clinical and molecular data of Rett patients, and it is currently one of the largest Rett registries worldwide with several hundred records provided by Rett expert clinicians from 13 countries. Collected data revealed that the majority of *MECP2*-mutated patients present with the classic form, the majority of *CDKL5*-mutated patients with the early-onset seizure variant, and the majority of *FOXG1*-mutated patients with the congenital form. A computation of severity scores further revealed significant differences between groups of patients and correlation with mutation types. The highly detailed phenotypic information contained in the Rett Networked Database allows the grouping of patients presenting specific clinical and genetic characteristics for studies by the Rett community and beyond. These data will also serve for the development of clinical trials involving homogeneous groups of patients.

## 1. Introduction

Rett syndrome (RTT, OMIM 312750) is a severe neurodevelopmental disorder that affects predominantly females with an incidence of approximately 1 in 10,000 female births mainly caused by mutations in the *MECP2* gene located in the X chromosome [1, 2]. Classic RTT is infrequently observed in males because a deleterious mutation in the only copy of *MECP2* typically results in severe neonatal encephalopathy and early lethality [3]. In the classic form, girls with RTT typically exhibit a relatively normal period of development for the first 6-18 months of life followed by a regression phase where patients lose acquired language and motor skills and exhibit intellectual disability and hand stereotypies. The hand stereotypies are typical in RTT and appear commonly to be continuous, located predominantly over the anterior body midline [4].

Beyond the classic form of RTT, a number of atypical forms with different degrees of severity have been described: the Zappella variant (formerly known as the preserved speech variant) [5, 6], the infantile seizure onset type [7], the congenital form [8], and the “forme fruste” [9]. Besides the *MECP2* gene, additional genes have been associated with the RTT phenotype. In particular, mutations in *CDKL5*, located on the X chromosome, have been reported in the infantile seizure onset type of RTT, while mutations in *FOXG1*, located on chromosome 14, have been reported in patients with the congenital presentation. It is still an object of debate if *CDKL5* and *FOXG1* mutations are responsible for atypical RTT or for a different neurodevelopmental phenotype [10–12].

Different RTT databases have been generated in the past and recent years. Among them are the International Rett Syndrome Association (IRSA) North American database and the InterRett [13, 14]. The Rett Networked Database (RND) is a registry of clinical and molecular data for patients affected by RTT and available at https://www.rettdatabasenetwork.org [15]. Although it was initially targeting the European population of patients with RTT, it is now open to countries outside of Europe. RND records are updated by clinicians with experience in RTT, limiting potential bias existing when clinical data are gathered using questionnaires sent out to families by mail. It is among the largest RTT registries worldwide with more than 1900 patients on file, and it is designed to be an open-access initiative since data can be retrieved directly through a web-based search engine by interested professionals upon the submission of a research proposal to the Scientific Review Board [15]. The public has access to general information and to content description while the individual patient file can be granted only upon registration of physicians and clinical researchers in charge of specific patients.

Here, we describe the first 1007 records contained in the registry and discuss the content of RND on the basis of the published guidelines for RTT clinical diagnosis [16, 17]. We analyzed the phenotype of patients with a *MECP2*, *CDKL5*, or *FOXG1* mutation to better understand the typical and atypical forms of RTT and provide information of RTT cohorts for the development of clinical trials.

## 2. Materials and Methods

### 2.1. RND Data

RND contains clinical files for 1958 patients affected by classic or atypical RTT (numbers are given as of March 1, 2017). Clinical data originates from Croatia (29 patients), Denmark (64 patients), France (252 patients), Hungary (82 patients), India (3 patients), Israel (93 patients), Italy (605 patients), Romania (15 patients), Serbia (50 patients), Spain (398 patients), United Kingdom (255 patients), USA (96 patients), and Russia (16 patients).

Patient clinical and genetic data were provided and inserted by the expert clinician through direct patients' evaluation, as described in Grillo et al. [15]. The system is able to collect 309 items (293 clinical and 16 genetic) grouped into 31 domains (30 clinical and 1 genetic). The system is permissive since patients with incomplete data can be inserted and later updated.

Data analysis is presented for the first 1007 patients aged over 5 years and for whom a pathogenic mutation in *MECP2*, *FOXG1*, or *CDKL5* has been identified. Enrolled patients either met the diagnostic criteria for RTT or had a mutation in *MECP2*. All participants had complete mutation testing including *MECP2* sequencing and deletion/duplication testing. Clinical diagnosis utilized the 2002 consensus criteria [12] or the revised diagnostic criteria for RTT published in 2010 [11]. *CDKL5*- and *FOXG1*-mutated patients were included whenever the diagnosis of RTT was achieved according to the 2002 consensus criteria or 2010 revised RTT criteria.

### 2.2. Data Analysis

Descriptive statistics were used to summarize the characteristics of the RND dataset. *MECP2* mutation types were grouped as Arg106Trp, Arg133Cys, Thr158Met, Arg306Cys, Arg168^∗^, Arg255^∗^, Arg270^∗^, Arg294^∗^, C-terminal deletion, early truncating mutations (mutations interrupting the MECP2 protein before amino acid 310), and large deletions. Those not falling in any of the above listed categories were grouped as “other.” *CDKL5* mutation types were clustered on the basis of early truncating mutations, late truncating mutations, large deletions, and missense mutations. *FOXG1* mutation types were grouped as early truncating mutations, late truncating mutations, gene deletions, and missense mutations.

Differences in clinical characteristics between groups of patients were tested by Fisher's exact test or by chi-squared analysis when the normal approximation was appropriate. R tool version 3.5.1 was used for statistical analyses, and *P* < 0.05 was considered as significant.

## 3. Results

### 3.1. Overview of RND Data

Among the 1007 RTT patients analyzed in this study, 806 were classified as classic, while the remaining 201 as atypical. Among this latter group, 46 had the congenital form of RTT, 36 patients the early-onset seizure variant, and 54 the Zappella variant (formerly known as the preserved speech variant). For the remaining 65 patients, the type of atypical form was not specified. All cases were sporadic except for 2 pairs of sisters and 5 pairs of monozygotic twins affected by RTT and carrying a *MECP2* mutation.

*MECP2* was mutated in 949 patients (94.2%), while 32 patients carried a mutation in *CDKL5* (3.2%) and 26 patients in *FOXG1* (2.6%).

### 3.2. Patients Carrying a Mutation in *MECP2*

Among the 949 *MECP2*-mutated patients, 804 have a diagnosis of classic RTT (84.7%), 24 the congenital variant (2.5%), five the early-onset seizure variant (0.5%), and 54 the Zappella variant (5.7%) and the remaining 62 have an atypical form of RTT not better specified in categories (6.5%). All mutation types are present in this population with p.Arg255^∗^, p.Thr158Met, and C-terminal deletions being the most frequent mutations despite significant difference between classic and atypical forms (Table 1).

Criteria for the clinical diagnosis of RTT were last revised in the RTT Diagnostic Criteria 2010 [11] in order to include a regression period, partial or complete loss of acquired purposeful hand skills, stereotypic hand movements, partial or complete loss of acquired spoken language, and gait abnormalities. We mined the RND data in order to investigate their compliance with the revised diagnostic criteria. Our analysis showed that, among the patients carrying a mutation in *MECP2*, regression occurred in 96.2% of patients, 86.5% lost or never acquired purposeful hand skills, 68.0% lost most or all spoken language, 68.1% had stereotypic hand movements, and 44.5% had gait dyspraxia (Table 2). On the other hand, the intense eye pointing phenotype of RTT patients is present in 87.6% of *MECP2*-positive cases (Table 2), although not included in the necessary criteria. In our dataset, the supporting criteria are present in about half of the patients carrying a mutation in *MECP2* (Table 2).

RND data were further interrogated to define the most frequent clinical signs of *MECP2* mutation carriers, among those retrieved in the RND (Table 3). This analysis revealed that, in addition to the necessary criteria for RTT diagnosis, a period of regression (96.2%), absence of speech (68.0%), a deficient sphincter control (88.5%), eye pointing (87.6%), feeding difficulties (85.2%), and a normal head circumference at birth (74.1%) are the main clinical signs in *MECP2-*mutated patients (Table 3).

Stereotypes, profound ID, and bruxism were present in 68.1%, 67.8%, and 62.1% of the study group, respectively. Fewer than one-third (28.0%) had never learned to walk independently. Epilepsy before 5 years of age was present in 63.0% of patients; in 3.9% of seizures, onset was before 1 year of age, and seizures were not controlled or barely controlled by therapy in 21.4% (Table 3).

**Figure 1:**
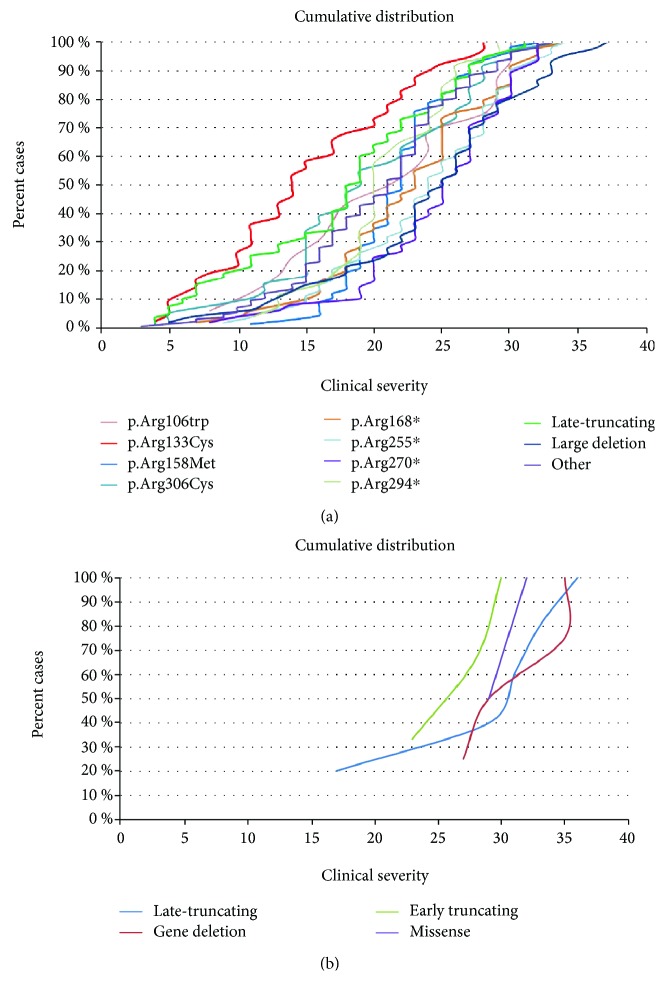
(a) Cumulative distribution plots of the patients positive for a *MECP2* mutation. Mutations were grouped based on Arg106Trp, Arg133Cys, Thr158Met, Arg306Cys, Arg168^∗^, Arg255^∗^, Arg270^∗^, Arg294^∗^, late truncating mutations (LTM), large deletions, and all other mutations. Early truncating mutations correspond to mutations interrupting the protein before amino acid 310. Large deletions correspond to deletions including either a single exon or the entire gene. (b) Cumulative distribution of the patients positive for a *FOXG1* mutation. Mutations were grouped as early truncating mutations (mutations interrupting protein before amino acid 275), late truncating mutations, gene deletions (deletions involving either single exons or the entire gene), and missense mutations.

A severity score was computed for the *MECP2*-mutated patients [6]. Although there is wide variability in clinical severity, there is a clear effect of specific common *MECP2* point mutations on median clinical severity. The cumulative distribution plots of patients positive for the *MECP2* mutations showed that the missense mutation Arg133Cys and late truncating mutations are associated to the less severe phenotype (Figure 1(a)). The missense mutations Arg306, Thr158, and Arg106 (arginine or threonine can be replaced by any amino acid) and the early truncating mutation Arg294^∗^ belong to the intermediate severity phenotype. The remaining early truncating mutations (Arg168^∗^, Arg255^∗^, and Arg270^∗^) and large deletions are associated with the “most severe” form of RTT syndrome (Figure 1(a)).

The cohort of *MECP2*-mutated RTT patients included also two pairs of sisters carrying the same *MECP2* mutation but with discordant clinical signs: one individual from each sibling pair could not speak or walk and had a profound intellectual deficit (classic RTT), while the other individual could speak and walk and had a moderate intellectual disability (Zappella variant). The five monozygotic twin pairs reported in RND were much more concordant than the sister pairs. Among the twin pairs, only two out of five had an identical clinical score, indicating that at least at this level of investigation, they were phenotypically identical. The remaining three twin pairs differed in specific fields such as epilepsy and weight (twin pair 1), level of speech and level of phrases (twin pair 2), or height, age of regression, and voluntary hand use (twin pair 3).

### 3.3. Patients Carrying a Mutation in *CDKL5*

RND contains 32 records for *CDKL5* mutation-positive cases. Thirty-one patients had a diagnosis of the early-onset seizure variant of RTT, while one was diagnosed as atypical RTT. The most frequent mutations, representing the 50% of *CDKL5*-mutated patients, were truncating mutations (28.1% of late truncating and 21.9% of early truncating mutations) followed by missense mutations (31.2%) and large deletion (18.8%). In our cohort, the majority of patients had a normal head circumference at birth (93.8%), a deficient sphincter control (96.0%), feeding difficulties (97.4%), IQ < 40 (100%), and presence of hand stereotypies (85.7%) and had never spoken (82.6%) (Table 3).

As for patients with *MECP2* mutations, it was possible to compute the total score for the *CDKL5*-mutated patients. However, no correlation was observed between type of mutation and clinical severity (data not shown).

### 3.4. Patients Carrying a Mutation in *FOXG1*

RND contained 26 records for *FOXG1* mutation-positive cases. Twenty-two patients had the congenital form of RTT, 2 patients had the classic form, and two patients were classified as atypical. The cumulative distribution of the patients positive for *FOXG1* mutations showed a clear trend toward a less severe phenotype for *FOXG1* late truncating mutations (Figure 1(b)). In our cohort, all patients carrying a *FOXG1* mutation had IQ < 40, microcephaly, and no speech at examination (Table 3).

### 3.5. Comparison among the Three Groups

Epilepsy before 5 years of age was statistically significant among groups of patients (*p* value 0.0001 *MECP2* vs. *CDKL5* and *p* value < 0.044 *MECP2* vs. *FOXG1*), since it was present in 63% of *MECP2*-mutated patients, in 96.9% (31 out of 32) of *CDKL5* cases, and in 87.5% of *FOXG1*-mutated patients. The epilepsy that started before 1 year of age was present in 96.9% of *CDKL5* patients with epilepsy, versus 3.9% of *MECP2-*mutated patients and 37.5% of *FOXG1-*mutated patients. Epilepsy was not controlled by therapy in 84% of *CDKL5*-mutated cases versus 21.4% of *MECP2*-mutated patients and 58.8% of *FOXG1*-mutated patients (Table 3).

Breathing dysfunction and eye pointing were statistically more frequent in *MECP2* patients (53.5% and 87.6%, respectively) rather than in *CDKL5* (11.5% and 40.6%, respectively) or *FOXG1* (28% and 13.3%, respectively) patients (Table 3). Conversely, motor and verbal disabilities were more severe in *CDKL5* and *FOXG1* patients rather than in *MECP2*-mutated patients. About 92.3% of patients carrying a *FOXG1* mutation had never spoken compared to the 59% of *MECP2* and 82.6% of *CDKL5*-mutated patients. Moreover, *FOXG1*-mutated patients had never learned to sit (78.3%) and walk (91.3%) compared to *MECP2* (7.7% and 28.0%, respectively) and *CDKL5* (23.1% and 74.1%, respectively).

Other features such as normal head circumference at birth, deficient sphincter controls, feeding difficulties, height and weight below the 25th percentile, troubled nighttime sleeping, and cold extremities were very similar among the three groups of patients carrying a *MECP2*, *CDKL5*, or *FOXG1* mutation (Table 3).

**Figure 2:**
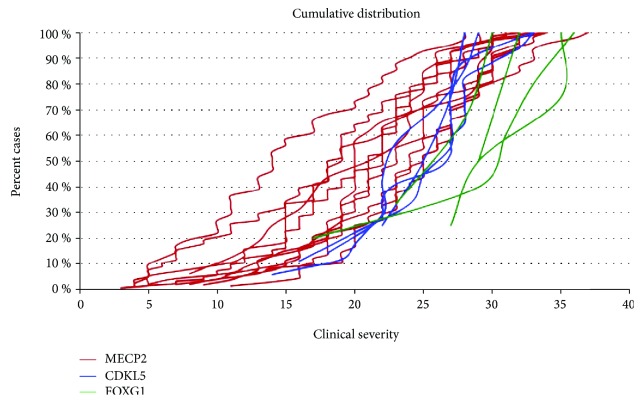
Combined graph illustrating the different clinical severities between *MECP2*-, *CDKL5*-, and *FOXG1*-mutated patient.

The overall cumulative distribution plot of patients carrying a mutation in the *MECP2*, *CDKL5*, or *FOXG1* genes is illustrated in Figure 2. *FOXG1* mutations confer the highest severity score, followed by *CDKL5* mutations. The majority of *MECP2* mutations are associated with the lowest severity score.

## 4. Discussion

Globally, the majority of RND patients do fulfill the necessary criteria for the diagnosis of RTT, according to the revised criteria [16]. A period of regression followed by recovery or stabilization, representing a required criterion for a diagnosis of RTT, is recorded in 96.2% of cases. The lack of recorded regression in nearly 4% of patients is probably due to the fact that in atypical RTT, especially in the congenital and early-onset seizure variants, the onset of neurological signs occurs in the first months of life, and in these cases, the regression is more difficult to ascertain.

Interestingly, although loss of acquired speech is included among RTT diagnostic criteria, RND data show that the majority of *MECP2*-positive cases have never spoken (59%), as reported in Table 3. Notably, hand stereotypies, although considered an invariant clinical sign of classic RTT, are absent in 31.9% of *MECP2*-mutated patients included in the RND dataset. It is however known that behind midline and exuberant hand stereotypies, many patients with *MECP2* may show more varied stereotypies or subtle stereotypes, like pill rolling or tapping [18]. Interestingly, although 85.2% of the *MECP2*-positive patients have feeding difficulties, only 43.3% have gastrointestinal disturbances. This would suggest that part of the feeding difficulties arise from abnormal muscle tone and oropharyngeal dysfunction [19]. Even though breathing mechanisms in RTT preclinical models have been heavily investigated, breathing dysfunction “only” affects 53.5% of the patients carrying a mutation in *MECP2* (Table 3). This is in line with a recent paper from the Rett Syndrome Natural History Study in which 51.6% of parents reported a history of hyperventilation, 67.1% a history of breath-holding, and 47.2% a history of air-swallowing during wakefulness [20].

Two earlier studies of the North American RTT Database relying on 915 patients with a mutation in *MECP2* were published [21, 22]. Similar to the Australian database, the data relies on questionnaires sent out to families, and even if the questionnaires were analyzed by experienced clinicians, the patients were not all directly examined by the contributors. Available results mainly concern molecular data with the distribution and nature of reported mutations. It does not contain *CDKL5* or *FOXG1* molecular data and does not provide details concerning the major phenotypic traits present in the studied population. In Kirby et al., 87.4% of patients with *MECP2* mutation have the typical form and 10.3% have the atypical form of RTT [22]. Similarly, the percentage of typical RTT patients with a *MECP2* mutation in the RND is 84.7%. The cumulative distribution showed that there is a wide clinical variability within the same *MECP2* mutation (Figure 1(a)). However, in accordance with previous reports [23, 24], the “mildest” mutations are Arg133Cys and late truncating mutations. The missense mutations Arg306, Thr158, and Arg106 (arginine or threonine can be replaced by any amino acid) and the early truncating mutation Arg294^∗^ belong to the intermediate severity phenotype. The remaining early truncating mutations (Arg168^∗^, Arg255^∗^, and Arg270^∗^) and large deletions are among the “most severe” form of RTT syndrome (Figure 1(a)). It is interesting to note that the plot of each mutation is not always parallel. For example, Thr158Met and Arg294^∗^ move more vertically, suggesting that the phenotype of patients who have these mutations is less influenced by other genetic or environmental factors.

Interestingly, the cohort of *MECP2*-mutated RTT patients included two pairs of sisters carrying the same *MECP2* mutation but with discordant clinical signs. One individual from each pair could not speak or walk and had a profound intellectual deficit (classic RTT), while the other individual could speak and walk and had a moderate intellectual disability (Zappella variant) [25].

The phenotype of the patients carrying a mutation in *CDKL5* and classified as having atypical RTT is much less documented than the classic RTT phenotype caused by *MECP2* mutations. A report in 2013 described 86 patients with a mutation in *CDKL5* with data originating from family questionnaires recorded in InterRett [26], and more recently, epilepsy and vagus nerve stimulation was studied in a cohort of 172 cases with a pathogenic CDKL5 mutation [27]. RND provided information in a cohort of 32 patients harboring a mutation in *CDKL5*. Expectedly, for the early seizure variant of RTT caused by *CDKL5* mutations, the majority of patients experienced at least one episode of epilepsy (>90% in all three cohorts). The proportion of patients with a mutation in *CDKL5* that never learned to walk in the three cohorts is also very similar (67.4% in InterRett, 64.6% in the International CDKL5 Disorder Database, and 74.1% in RND), together with the proportion of patients displaying hand stereotypies (80.3% of females in InterRett and 85.72% of patients positive for a mutation in *CDKL5* in RND) [26, 27]. There is a difference between the two cohorts concerning the speech skills, since 30 out of 76 females (39.5%) with *CDKL5* mutation acquired early speech skills in the InterRett cohort and 39/172 (22.7%) had the simplest level of communication in the International *CDKL5* Disorder Database while only 17.4% females harboring a *CDKL5* mutation had shown a somewhat level of speech in RND [26, 27].

Regarding CDKL5-mutated patients, no significant genotype-phenotype correlation was observed. The phenotype of the patients carrying a mutation in *FOXG1* and classified as having atypical RTT is even less documented than the phenotype caused by *CDKL5* mutations. The cumulative distribution in Figure 1(b) shows a clear trend toward a less severe phenotype for *FOXG1* late truncating mutations. The cumulative overall distribution in Figure 2 nicely illustrates the progressive severity going from *MECP2* to *CDKL5* and *FOXG1* mutation. *CDKL5* patients lie in the most severe range in comparison to *MECP2* patients with *FOXG1* patients even more shifted than *CDKL5* patients towards a worse clinical phenotype and a very minimum overlap with *MECP2* patients.

**Figure 3:**
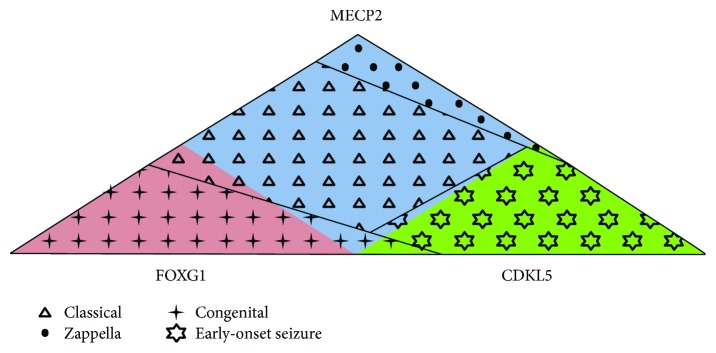
Genotypes and phenotypes in RND. The majority of *MECP2*-mutated patients (light blue) have the classic form (triangles), the majority of *CDKL5*-mutated patients (green) have the early-onset seizure variant (stars), and the majority of *FOXG1*-mutated patients (pink) have the congenital form (crosses). Several exceptions to this rule are present: among the *MECP2*-mutated patients (light blue), about 6% has the Zappella variant (dots), about 2.5% has the congenital variant of RTT (crosses), and about 0.5% has the early-onset seizure variant (stars); among the *CDKL5*-mutated patients (green), about 3% of the patients has other atypical forms of RTT (dots and crosses); and among the *FOXG1*-mutated patients (pink), about 7% of patients has the classic form (triangles).

In conclusion, the Rett Networked Database is a registry for patients with RTT where clinical data are validated by experienced clinicians upon direct examination of the affected individuals. One of the unique features of this database is its ability to collect a huge amount of clinical details, the collected clinical items being almost 300 with different levels of completeness, and genetic data [10–15]. RND collects data from 13 different countries; however, at the moment, it could not be considered representative of all the countries from which data is sourced given the different involvement of each country in terms of shared entries. Its strength is that it contains a large number of cases, thus providing a powerful resource to perform genotype-phenotype correlations of RTT patients from European countries and beyond. Overall, observation of RND data highlights clinical characteristics which occur more frequently in patients with a specific mutation (Table 3). For example, presence of regression and gait dyspraxia are statistically more frequent in *MECP2*-mutated patients; epilepsy and reduction in eye pointing capability are statistically more frequent in *CDKL5*-mutated patients, while the large majority of *FOXG1* patients have never learned to walk, sit, and speak. Moreover, we observed that the majority of *MECP2*-mutated patients have the classic form of RTT, the majority of *CDKL5*-mutated patients have the early-onset variant, and the majority of *FOXG1*-mutated patients have the congenital form, with some exceptions (Figure 3). RND provides an open structure, available to all interested professionals, and a searchable web interface made available for registered users. These characteristics should prove useful to perform additional phenotype-genotype correlations, to better understand the typical and atypical forms of RTT, and to select adequate patient populations for future clinical trials.

## Figures and Tables

**Table 1:** *MECP2* patients classified as classic or atypical RTT according to the mutation types reported in RND (total number of patients 949).

Mutation type	Total (%) (*n* = 949)	Classic RTT *N* (%) (*n* = 804)	Atypical RTT *N* (%) (*n* = 145)	*p* value classic vs. atypical RTT
p.Arg106Trp	31 (3.3)	26 (83.9)	5 (16.1)	0.894
p.Arg133Cys	62 (6.5)	37 (59.7)	25 (40.3)	**<0.0001**
p.Thr158Met	102 (10.8)	91 (89.2)	11 (10.8)	0.182
p.Arg168^∗^	80 (8.4)	74 (92.5)	6 (7.5)	**0.043**
p.Arg255^∗^	106 (11.2)	96 (90.6)	10 (9.4)	0.076
p.Arg270^∗^	62 (6.5)	57 (91.9)	5 (8.1)	0.102
p.Arg294^∗^	63 (6.6)	59 (93.7)	4 (6.3)	**0.041**
p.Arg306Cys	67 (7.1)	58 (86.6)	9 (13.4)	0.663
C-Terminal deletion	101 (10.6)	78 (77.2)	23 (22.8)	**0.027**
Early truncating	93 (9.8)	80 (86)	13 (14)	0.713
Large deletion	72 (7.6)	65 (90.3)	7 (9.7)	0.173
Other	110 (11.6)	83 (75.4)	27 (24.5)	**0.004**

*N*: number of cases for which the corresponding item is present; percentage is provided in brackets; the *p* value of significance is provided for comparison.

**Table 2:** Compliance of the RND data with the revised diagnostic criteria [11] for patients positive for a mutation in *MECP2*. Peripheral vasomotor disturbances are accounted for in the “small cold hands and feet” score. The item “diminished response to pain” is not present in RND data.

Clinical sign	*N*	*N*+ (%)
A period of regression	743	715 (96.2)
*Necessary criteria*		
Partial or complete loss of acquired purposeful hand skills	743	673 (86.5)
Stereotypic hand movements	880	599 (68.1)
Partial or complete loss of acquired spoken language	754	513 (68.0)
Gait abnormalities	821	365 (44.5)
*Supportive criteria*		
Breathing disturbances when awake	824	441 (53.5)
Bruxism when awake	829	515 (62.1)
Impaired sleep pattern	926	419 (45.2)
Abnormal muscle tone	—	—
Peripheral vasomotor disturbances	—	—
Scoliosis or kyphosis	853	444 (52.1)
Growth retardation^∗^	771	419 (54.3)
Small cold hands and feet	160	81 (50.6)
Inappropriate laughing or screaming spells	560	171 (30.5)
Diminished response to pain	—	—
Eye pointing	958	843 (88.0)

*N* represents the number of cases for which the corresponding item is present in the patient file; *N*+ represents the number of cases positive for the clinical signs, and the percentage is provided in brackets. ^∗^Growth retardation was considered to be present when the weight was below the 25th percentile. When height is considered, 54.3% of *MECP2*-positive patients are below the 25th percentile.

**Table 3:** Main clinical characteristics in patients positive for a mutation in *MECP2*, *CDKL5*, and *FOXG1*. Clinical characteristics are listed in descending order of percentage of patients harboring a mutation in *MECP2*.

Clinical sign	*MECP2* (*N* = 949)		*CDKL5* (*N* = 32)		*FOXG1* (*N* = 26)		*p* value *MECP2* vs. *CDKL5*	*p* value *MECP2* vs. *FOXG1*	*p* value *CDKL5* vs. *FOXG1*
	*N*	*N*+ (%)	*N*	*N*+ (%)	*N*	*N*+ (%)			
A period of regression	743	715 (96.2)	16	12 (75.0)	25	13 (52.0)	**<0.0001**	0.140	**<0.0001**
Normal head circumference at birth	769	570 (74.1)	32	30 (93.8)	17	14 (82.4)	**0.012**	0.447	0.210
Deficient sphincter control	736	651 (88.5)	25	24 (96.0)	23	22 (95.7)	0.241	0.283	0.952
Eye pointing	880	771 (87.6)	32	13 (40.6)	15	2 (13.3)	**<0.0001**	**<0.0001**	0.052
Feeding difficulties	813	693 (85.2)	38	37 (97.4)	15	13 (86.7)	**0.0364**	0.877	0.128
Presence of hand stereotypies	880	599 (68.1)	28	24 (85.7)	24	23 (95.8)	**0.048**	**0.004**	0.217
IQ < 40	704	477 (67.8)	19	19 (100)	24	24 (100)	**0.003**	**0.0008**	1
Microcephaly or deceleration of head growth	771	419 (54.3)	27	12 (44.4)	26	26 (100)	0.310	**<0.0001**	**<0.0001**
Gait dyspraxia	821	365 (44.5)	27	20 (74.1)	21	2 (9.5)	**0.002**	**0.001**	**<0.0001**
No speech at examination	754	513 (68.0)	23	21 (91.3)	24	24 (100)	**0.018**	**0.0009**	0.140
Epilepsy before 5 years of age	548	345 (63.0)	32	31 (96.9)	16	14 (87.5)	**0.0001**	**0.044**	0.206
Scoliosis	853	444 (52.1)	24	2 (8.3)	22	6 (27.3)	**<0.0001**	**0.022**	0.091
Bruxism	829	515 (62.1)	32	14 (43.8)	17	11 (64.7)	**0.036**	0.828	0.163
Height below the 25th percentile	767	447 (58.3)	27	13 (48.1)	25	17 (68.0)	0.295	0.332	0.148
Cold extremities	160	81 (50.6)	3	1 (33.3)	22	10 (45.5)	0.553	0.649	0.692
Weight below the 25th percentile	771	419 (54.3)	24	10 (41.7)	25	17 (68.0)	0.220	0.177	0.064
Has never spoken	754	445 (59.0)	23	19 (82.6)	26	24 (92.3)	**0.023**	**0.0007**	0.301
Gastrointestinal disturbances	603	261 (43.3)	26	10 (38.5)	22	16 (72.7)	0.627	**0.006**	**0.018**
Breathing dysfunction	824	441 (53.5)	26	3 (11.5)	25	7 (28.0)	**<0.0001**	**0.012**	0.139
Troubled night time sleeping	926	431 (46.6)	32	14 (43.8)	18	11 (61.1)	0.756	0.220	0.239
Never learned to walk	821	230 (28.0)	27	20 (74.1)	23	21 (91.3)	**<0.0001**	**<0.0001**	0.114
Epilepsy not controlled by therapy	548	117 (21.4)	25	21 (84.0)	17	10 (58.8)	**<0.0001**	**0.0003**	0.069
Never learned to sit	664	51 (7.7)	26	6 (23.1)	23	18 (78.3)	**0.005**	**<0.0001**	**0.0001**
Epilepsy before 1 year of age	689	27 (3.9)	32	31 (96.9)	16	6 (37.5)	**<0.0001**	**<0.0001**	**<0.0001**

*N* represents the number of cases for which the corresponding item is present in the patient file; *N*+ represents the number of cases positive for the clinical sign, and the percentage is provided in brackets; the *p* value of significance is provided for comparison.

## Data Availability

Data are available at https://www.rettdatabasenetwork.org/.
